#  Correction

**DOI:** 10.1111/cas.15711

**Published:** 2023-02-15

In an article^1^ titled “Adjuvant atezolizumab in Japanese patients with resected stage IB‐IIIA non‐small cell lung cancer (IMpower010)” by Hirotsugu Kenmotsu, Shunichi Sugawara, Yasutaka Watanabe, Haruhiro Saito, Morihito Okada, Toyofumi Fengshi Chen‐Yoshikawa, Yuichiro Ohe, Wataru Nishio, Shizuka Nakagawa, Haruka Nagao, there were errors in the heading of Figure 3.

The revised Figure 3 is shown below:

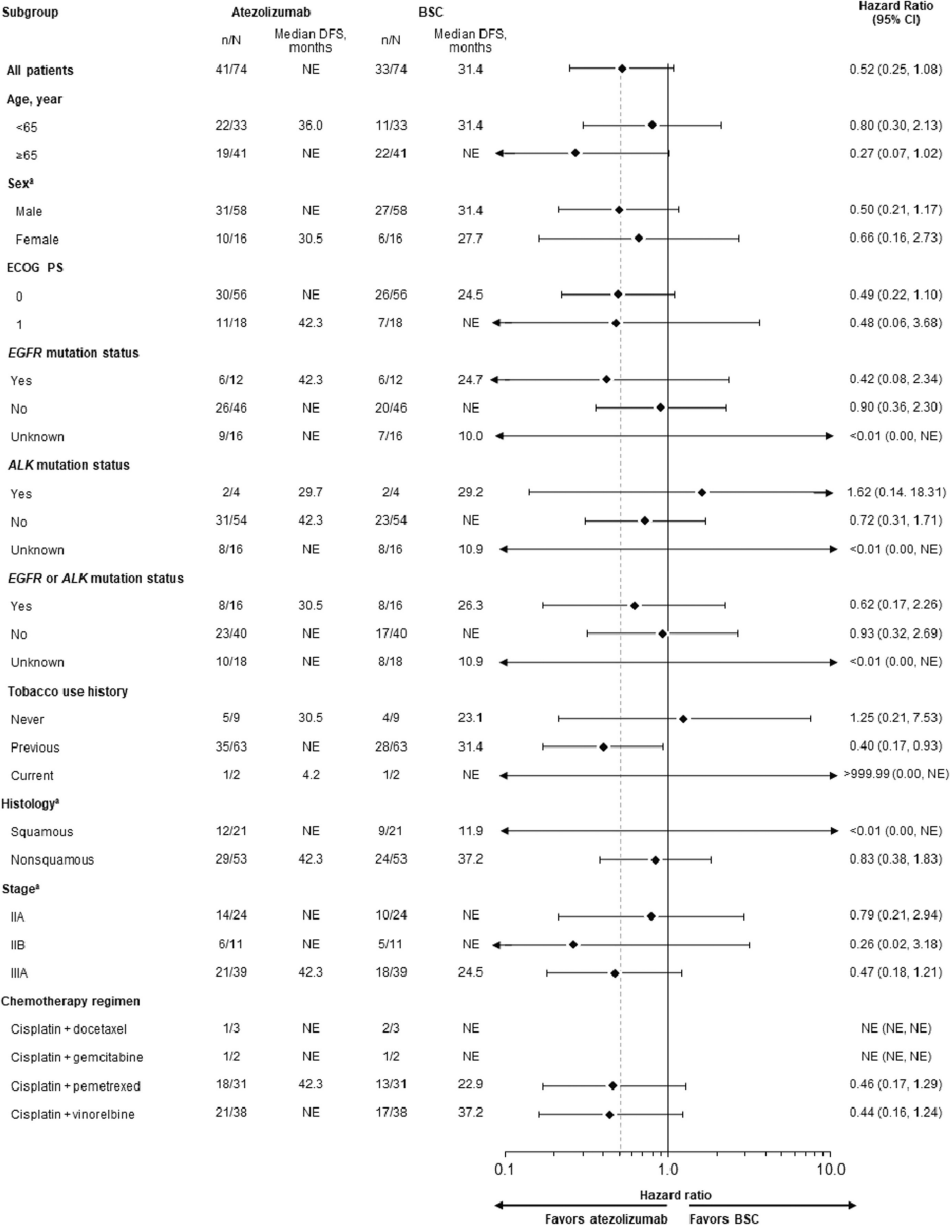

The authors apologize for the errors.
